# Spatial and Temporal Patterns of Apparent Electrical Conductivity: DUALEM *vs.* Veris Sensors for Monitoring Soil Properties

**DOI:** 10.3390/s140610024

**Published:** 2014-06-06

**Authors:** João Serrano, Shakib Shahidian, José Marques da Silva

**Affiliations:** University of Évora, ICAAM, Apartado 94, 7002-554 Évora, Portugal; E-Mails: shakib@uevora.pt (S.S.); jmsilva@uevora.pt (J.M.S.)

**Keywords:** apparent soil electrical conductivity, DUALEM sensor, soil cover vegetation, soil properties, Veris

## Abstract

The main objective of this study was to compare two apparent soil electrical conductivity (EC_a_) sensors (Veris 2000 XA and DUALEM 1S) for mapping variability of soil properties in a Mediterranean shallow soil. This study also aims at studying the effect of soil cover vegetation on the EC_a_ measurement by the two types of sensors. The study was based on two surveys carried out under two very different situations: in February of 2012, with low soil moisture content (SMC) and with high and differentiated vegetation development (non grazed pasture), and in February of 2013, with high SMC and with short and relatively homogeneous vegetation development (grazed pasture). The greater temporal stability of Veris sensor, despite the wide variation in the SMC and vegetation ground cover indicates the suitability of using this sensor for monitoring soil properties in permanent pastures. The survey carried out with the DUALEM sensor in 2012 might have been affected by the presence of a 0.20 m vegetation layer at the soil surface, masking the soil properties. These differences should be considered in the selection of EC_a_ sensing systems for a particular application.

## 1. Introduction

Southern Portugal presents significant areas with shallow soils, degraded through erosion and loss of nutrients as a consequence of many decades of intensive cereal monoculture. High spatial variability is usually found in the soil due to many physical, biological and chemical processes acting simultaneously [1,2]. Intensive grid-sampling is generally regarded as one of the most accurate means of evaluating this spatial variability. Taking into account the need for many soil samples in order to achieve a good representation of any soil property, the traditional method of soil sampling, mapping approaches and laboratory work is costly, labour-intensive and not feasible at a real farm scale [3,4]. Therefore, it is desirable to find other, more rapid means of obtaining information for detailed soil mapping [3]. Interest in management of field variability has increased with the increasing availability and adoption of precision agriculture tools and technology. The application of geospatial measurements of EC_a_ combined with the use of global navigation satellite systems (GNSS) and geographical information systems (GIS) is one of the most reliable techniques to characterize the spatial pattern of soil properties within fields [2,5–9]. As well as providing a correlation with soil properties, soil electrical conductivity mapping can also be used for delineating management zones and soil boundaries [2,10].

There are two types of commercially available electrical conductivity sensors for measuring soil EC_a_ in the field: contact and non-contact sensors [11]. The earliest sensors were of the contact type (for example, the Veris sensor): they introduce an electrical current into the soil through current electrodes, usually in the shape of coulters that make contact with the soil to directly measure the soil resistance or electrical resistivity (ER); the difference in current flow potential is measured at potential electrodes that are placed in the vicinity of the current flow [5,7]. The non-contact or non-invasive soil sensors (for example, the DUALEM sensor) are based on the principle of electromagnetic induction (EMI) and, presently, these are the most commonly used for sensing techniques [12]. A transmitter coil located at one end of the EMI instrument induces eddy-current loops in the soil with the magnitude of these loops directly proportional to the electrical conductivity in the vicinity of that loop. Each current loop generates a secondary electromagnetic field that is proportional to the value of the current flowing within the loop. A fraction of the secondary induced electromagnetic field from each loop is intercepted by the receiver coil of the instrument and the sum of these signals is amplified and formed into an output voltage which is related to a depth-weighted soil electrical conductivity [7,13].

Each of the two different methods to obtain EC_a_ has operational advantages and disadvantages [5]. The ER sensors, such as Veris, are usually much heavier and require a tractor or truck to pull them through the field, limiting their use to firmer soil conditions and unplanted fields [5]. This type of sensors require good contact between the soil and electrodes inserted into the soil; consequently, they produce less reliable measurements in dry or stony soils than the non-invasive measurement [7]. Using a wheeled cart pulled by an all-terrain vehicle, EMI sensors are generally adaptable to a wide variety of data collection conditions. These systems are very light, which means that they requires little drawbar pull allowing them to collect data under any soil moisture conditions, without traffic lines problems in situations of near field capacity. Also, it is possible to collect data after a crop has been planted up until the time that the crop is 15 to 20 cm tall. According to Corwin and Lesch [14], the use of mobile EMI equipment has three advantages over the use of mobile ER equipment: (i) the ability to take measurements on dry and stony soils, (ii) the ability to traverse growing crops, and (iii) the ability to traverse fields with beds and furrows. For Abdu *et al.* [13] an additional advantage of the EMI sensors is that they can be used to characterize soil spatial variability across large areas, due to their higher operating speeds.

As the use of EC_a_ sensing in precision agriculture becomes more generalized, it will be important to compare the data obtained with each type of system and to understand how these data are related to soil properties [5]. Others studies documented the comparison between different sensors used for measuring EC_a_. For example, Abdu *et al.* [13], Urdanoz *et al.* [15] and Saey *et al.* [16] compared two EMI sensors (the DUALEM 1S with the Geonics EM38DD; the DUALEM 1S with the Geonics EM38RT; and the DUALEM 21S with the EM38DD, respectively); Sudduth *et al.* [5] and Fulton *et al.* [17] compared EC_a_ measurements from a non-contact, electromagnetic induction-based sensor (Geonics EM38) to those obtained with a contact sensor (Veris 3100).

Previous studies by the authors [18,19] demonstrate the interest in using the DUALEM 1S sensor (EMI sensor) for monitoring the soil, and showed on the one hand a strong positive correlation between EC_a_ and SMC, and on the other, the temporal stability of EC_a_ patterns under different soil moisture contents (11.9 ± 1.0% in dry conditions and 23.9 ± 1.8% in wet conditions). Nonetheless, these works, unlike what has been shown by the studies of Sudduth *et al.* [5], Abdu *et al.* [13] and Brevik *et al.* [20] in terms of relation between EC_a_ data and soil properties, did not reveal significant correlations between EC_a_ and soil attributes that remain relatively stable throughout the years, such as clay and organic matter. According to Sudduth *et al.* [5] and King *et al.* [3], in saline soils the biggest contributor to electrical conductivity is the solute concentration, but for most temperate soils (where salt concentrations are small) the major influences are moisture and clay content. In non-saline soils with low concentrations of dissolved electrolytes, EC_a_ is highly correlated to SMC. In this type of soils, the pattern of EC_a_ reflects, in particular, the SMC, which interferes with the effect of clay or organic matter on the EC_a_. Other factors that have been mentioned as partially influencing this correlation between the EC_a_ and the clay or organic matter in shallow soils are the horizon depth dynamics (the bedrock depth and weathered bedrock) and the amplitude of clay and organic matter spatial variability [19].

The issue of ground cover at the moment of EC_a_ survey has been mentioned by Corwin and Lesch [14] as being elusive and one of the difficulties associated with ER sensors, and has been studied by Sam and Ridd [21] and Brevik *et al.* [22]. These researchers sought to identify differences in readings taken by EC_a_ sensors above crop residues or in soils without crop residues, but no works have been published yet with fully developed crops at the time of EC_a_ surveys.

Given these results, the main objective of this study was to compare two types of EC_a_ sensors: the contact ER sensor (Veris 2000 XA) and non-contact EMI sensor (DUALEM 1S) for mapping soil properties variability (bedrock depth, SMC, texture, pH, organic matter and macronutrients), in a Mediterranean shallow soil and to clarify if this correlation (EC_a_ *vs.* soil properties) in pastures is specific to the EMI sensor or it is similar to ER sensors. This study aims at studying the effect of vegetation on the EC_a_ measurement by the two types of sensors, carrying out the trials under two very different situations: with high vegetation and differentiated development (non grazed pasture) and with short vegetation and relatively homogeneous development (grazed pasture).

## 2. Material and Methods

### 2.1. Site Characteristics

The experimental field, with an area of about 6 ha, is located at the Revilheira farm (38°27′51.6″N and 7°25′46.2″W) in Southern Portugal. The predominant soil of the field is classified as a Leptic Luvisol [23]. The Leptic Luvisol profile is characterized by a pedogenetic differentiation of clay content with a lower content in the topsoil (0.15 to 0.50 m) and a higher content in the subsoil. Luvisols on steep slopes are very prone to erosion in regions with distinct dry and wet seasons, such as the Mediterranean region, where the soils of the upper slopes are usually more shallow due to many years of deep cultivation for intensive cereal production. In this region these shallow soils are used mainly for extensive grazing or planted to tree crops.

Topography affects soil characteristics, such as texture and thickness, which in turn condition the soil electric conductivity. A topographic survey of the area was carried out using a Real Time Kinematic (RTK) GPS instrument (Trimble RTK/PP-4700 GPS, Trimble Navigation Limited, Sunnyvale, CA 94085, USA). The elevation data were sampled in the field with the GPS assembled on an all-terrain vehicle. The digital elevation model surface was created using the linear interpolation TIN tool from ArcGIS 9.3 and converted to a grid surface with a 1 m grid resolution.

The bedrock depth of the experimental field was determined using a pneumatic gouge auger in twenty geo-referenced samples (56 m × 56 m grid). The depths were organized into four classes, namely: 0–0.20 m; 0.20–0.30 m; 0.30–0.50 m; and more than 0.50 m.

### 2.2. Soil and Pasture Sample Collection and Analysis

Soil spatial variability of the 6 ha experimental field was characterized by seventy-six samples geo-referenced with GPS taken in February of 2012 and 2013, from the whole parcel (one from each 28 × 28 m square). The soil samples were collected using a gouge auger and a hammer, in a depth range of 0–0.30 m. The soil was characterized in terms of texture, moisture content, pH, organic matter content and macronutrients (nitrogen, phosphorus and potassium). Each composite sample was the result of five sub-samples, one taken from the center of the square, and the other four taken near the corners of the square. The soil samples were kept in plastic bags, air-dried and analysed for particle-size distribution using a sedimentographer (Sedigraph 5100, manufactured by Micrometritics, Norcross, GA 30093-2901, USA), after passing the fine components through a 2 mm sieve. These fine components were also analysed for pH in 1:2.5 (soil:water) suspension, using the potenciometric method. Organic matter was measured by combustion and CO_2_ measurement, using an infrared detection cell. The NO_3_ was measured using the selective ion method. P_2_O_5_ and K_2_O were extracted by the Egner-Riehm method, and P_2_O_5_ was measured using colorimetric method, while K_2_O content was measured with a flame photometer. The soil samples were weighed, dried at 70 °C for 48 h, and then weighed again to establish the SMC.

Twenty geo-referenced pasture samples of 1 m^2^ areas from each field square were collected in February of 2012, using manual shears. The collected samples were stored in marked plastic bags and taken to the Pasture and Forage Laboratory of the University of Évora. These samples were weighed to determine the green matter production per hectare, and subsamples in small paper bags were placed in a 65 °C oven for 48 h to determine the pasture moisture content, which was used to calculate pasture dry matter yield. In February 2013, no pasture samples were collected as animal grazing resulted in short vegetation with relatively homogeneous development.

### 2.3. Apparent Soil Electrical Conductivity Surveys

**Figure 1.**
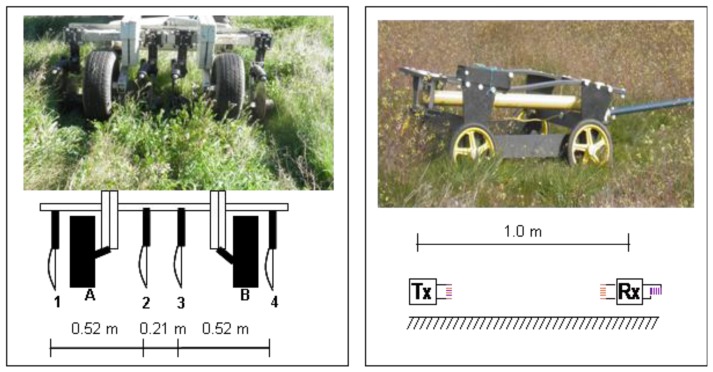
Above: survey with Veris 2000 XA (**left**) and DUALEM 1S (**right**) sensors in the experimental field (February 2012); bottom: geometry of Veris 2000 XA (left; A and B are wheels and 1–4 are rotating discs or coulter-electrodes) and DUALEM 1S sensor (right: Tx is the transmitter and Rx the two receivers).

The Veris 2000 XA contact-type sensor (Veris Technologies, Salina, KS, USA) and the DUALEM 1S non-contact sensor (Dualem, Inc., Milton, ON, Canada), equipped with a global positioning system (GPS) antenna were used to measure the apparent soil electrical conductivity in the experimental field (Figure 1).

The Veris 2000 XA soil resistance sensor (Figure 1, left) is mounted on a chassis supported on two wheels and is formed by two pairs of Coulter-electrodes (rotating discs), adjustable from 0.61 m to 0.91 m: one pair injects a current into the soil (outermost discs, 1 and 4), while the other pair of coulter-electrodes (the two innermost discs, 2 and 3) measure the voltage drop. Thus, this sensor generates one set of topsoil data, weighted depth readings, from 0 to 0.30 m pseudo depths (PD). The sensor was pulled by a conventional tractor.

The DUALEM-1S sensor (Figure 1, right) has 1 m separation between its transmitter (Tx) and two receivers (Rx), so its dual depths of conductivity measurement are 0–0.50 m and 0–1.50 m depth, with 70% of the cumulative influence of the EC_a_ evaluated by the sensor coming from the top 0–0.50 m and 0–1.50 m soil layers, respectively [24]. Due to the sensitivity of this sensor to metallic structures, a four-wheel vehicle equipped with a 3 m long tow arm was built from PVC, and pulled by a pickup truck. Given the height of the vehicle (0.20 m) and assuming that the EC_a_ of the air is zero [10], the EC_a_ readings were effectively made from the top 0–0.30 m and 0–1.30 m PD.

**Figure 2.**
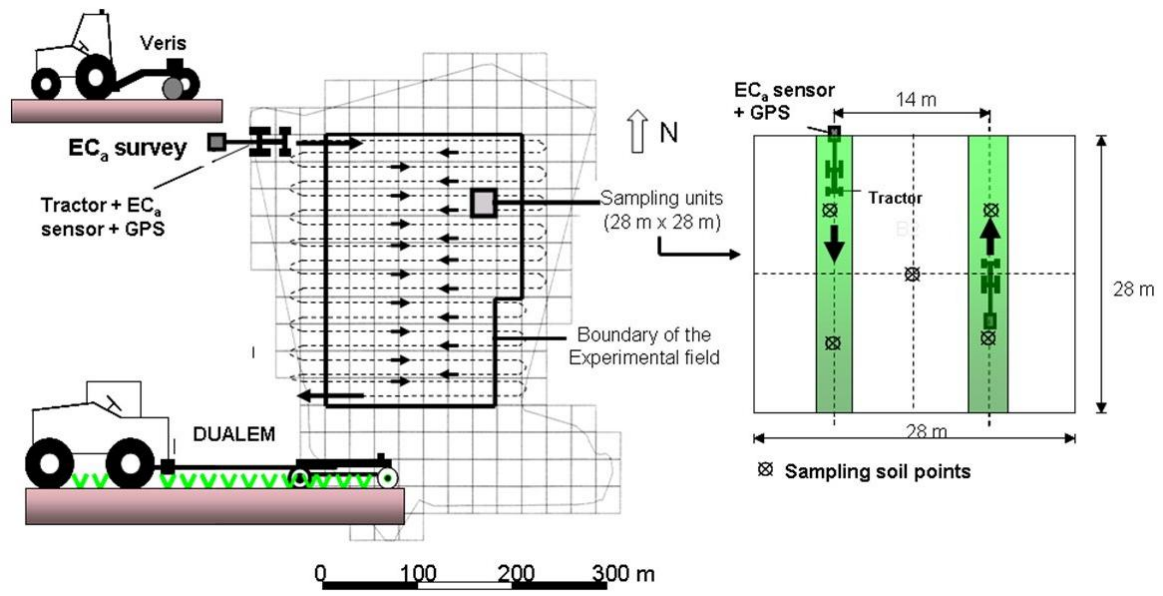
Diagram illustrating the procedure used to survey the soil electrical conductivity (**left**) and to collect soil sampling in each square (**right**) in the experimental field.

Both EC_a_ surveys were conducted sequentially in February (2012 and 2013) on the same day, with negligible temperature differences. Both sensors were towed at an average speed of 5 km·h^−1^. Each 28 m by 28 m square was covered twice in opposite directions, with a spacing of about 14 m (Figure 2). The electrical conductivity sensors were programmed to register the measurements each second.

The surface maps of soil parameters were developed in ArcGIS 9.3, using a 5 m grid inverse squared-distance interpolator which was subsequently average filtered with a 5 m × 5 m mesh grid.

### 2.4. Statistical Treatment

The EC_a_ data were analysed in two steps. In the first step, to allow comparison between Veris and DUALEM sensors, the data obtained in the two years of the experiment (2012 and 2013) were synchronized using the geographic co-ordinates of each point. A combined data set was created in each year: each Veris data point was combined with the nearest DUALEM data point based on GPS co-ordinates. If a match in spatial co-ordinates was not found within a 2 m radius, that point was removed from the data set, a procedure similar to that used by Sudduth *et al.* [5]. A total of 964 points out of 2109 were found to have common geographic co-ordinates in both surveys. These set of data were subjected to linear correlation analysis, carried out in 20.0 SPSS Statistical Package for Windows (SPSS Inc., Chicago, IL, USA) to obtain the Pearson correlation coefficients (r) using the method of minimum squares (*p* < 0.05). In the second step, the EC_a_ data obtained by Veris sensor and the data obtained by DUALEM sensor only from the top 0–0.30 m PD were used. In processing the data, using geographic co-ordinates as the basis, average electrical conductivity values were obtained using the values registered in each 28 m by 28 m square. In practice, the average value of electrical conductivity of each square was obtained using the arithmetic average of 28–30 registered values. Linear correlation analysis between the average EC_a_ data (obtained by the Veris and DUALEM sensors) from the 0–0.30 m PD and the different soil variables (in 2012 and 2013) and the pasture dry matter and pasture moisture content (in 2012) were also carried out. This methodology is similar to that used by Moral *et al.* [2] and Sudduth *et al.* [5].

## 3. Results and Discussion

### 3.1. Soil Spatial Variability

**Figure 3.**
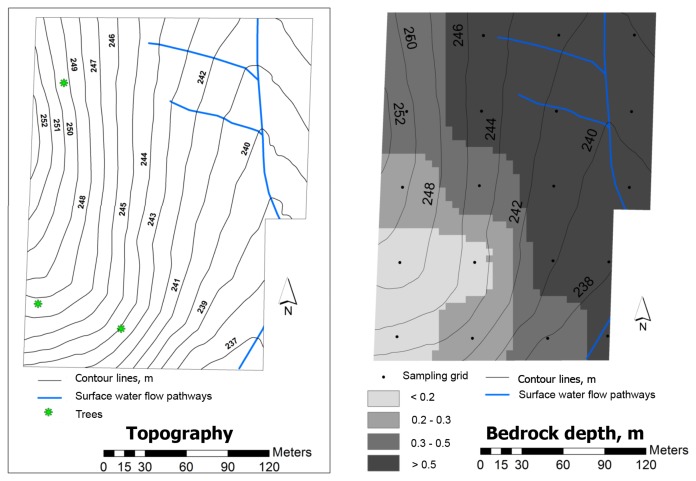
Topography map (**left**) and bedrock depth (**right**) of the experimental field [25].

Figure 3 shows the topography and the bedrock depth maps of the experimental field. At the lower positions and in the slope areas, the typical bedrock depths are more than 0.50 m, with a tendency for smaller depths (<0.20 m) at the higher elevations.

Table 1 presents the range, mean and standard deviation of apparent soil electrical conductivity and other soil parameters of the experimental field. The soil of the experimental field has clay loam texture, low levels of organic matter (<3%), slightly acid pH, high levels of potassium, and low levels of nitrogen and phosphorus.

The results show that the EC_a_ measured by DUALEM sensor are much higher than those measured by Veris sensor in 2012, when the soil had lower moisture content (10.7 ± 2.4%), but taller vegetation with differentiated development (non-grazed pasture). In 2013, when the soil had high moisture content (21.5 ± 2.4%), besides a clear decrease in the values measured by the DUALEM sensor (changed from 66.1 ± 4.5 mS·m^−1^ in 2012 to 20.0 ± 8.6 mS·m^−1^ in 2013 in the 0–0.30 m PD, and from 76.3 ± 2.3 mS·m^−1^ in 2012 to 30.6 ± 9.4 mS·m^−1^ in 2013 in the 0–1.30 m PD) a slight increase in the values measured by Veris sensor (changed from 4.0 ± 1.6 mS·m^−1^ in 2012 to 6.8 ± 4.5 mS·m^−1^ in 2013) was also observed.

**Figure 4.**
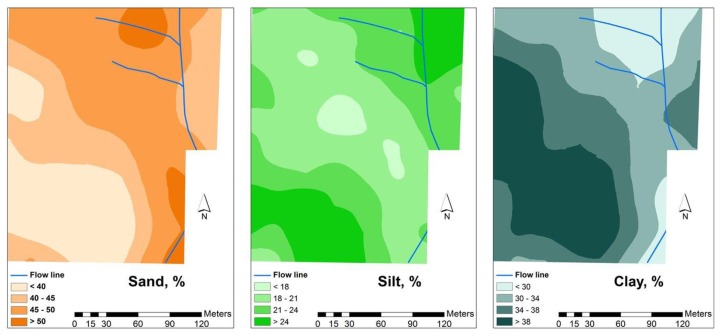
Sand, silt and clay content in the soil of the experimental field [25].

**Figure 5.**
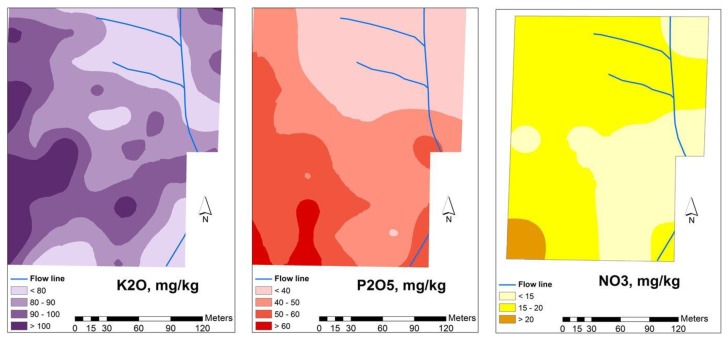
Macronutrients (K_2_O, P_2_O_5_ and NO_3_) in the soil of the experimental field [25].

**Figure 6.**
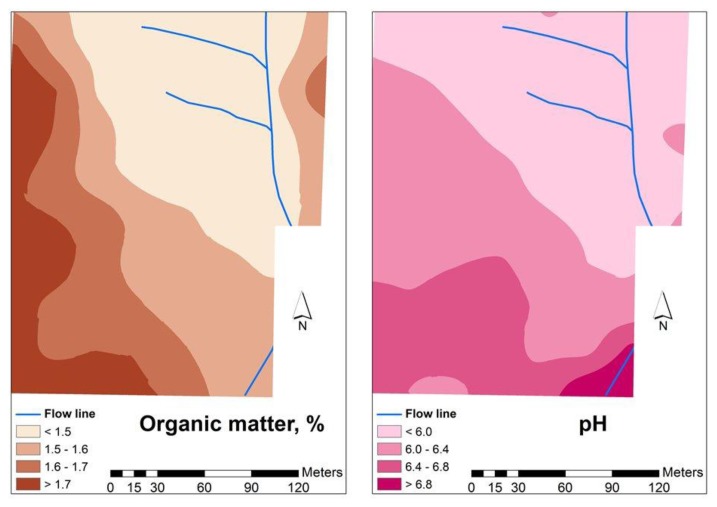
Organic matter content and pH in the soil of the experimental field [25].

Figures 4, 5 and 6 show the spatial variability of soil properties from experimental field (average of values measured in 2012 and 2013). The patterns of clay are complementary of those of sand, with higher rates of clay in the south-west areas of the field and lower rates of clay in the north-east. Silt, has an intermediate spatial pattern. These maps also show that the experimental field is dominated by a large spatially heterogeneous trend. The landscape topography has caused many of the studied soil parameters to vary, a phenomenon which was also observed by Kumhálová *et al.* [26] and Guo *et al.* [27]. If, on the one hand, the occurrence of higher concentration of soil organic matter in the south-west areas (upper areas) of grazed pasture field is justified, since these are areas where the trees are located and the animals have a tendency to spend more time, resulting in a concentration of dung deposition, on the other, the higher rates of soil clay content in the upper areas of the field show, in these shallow soils, that the B horizon is exposed or very near the soil surface in the eroded areas of the landscape, while in the valleys, this same clay horizon is located at a greater depth [18].

**Figure 7.**
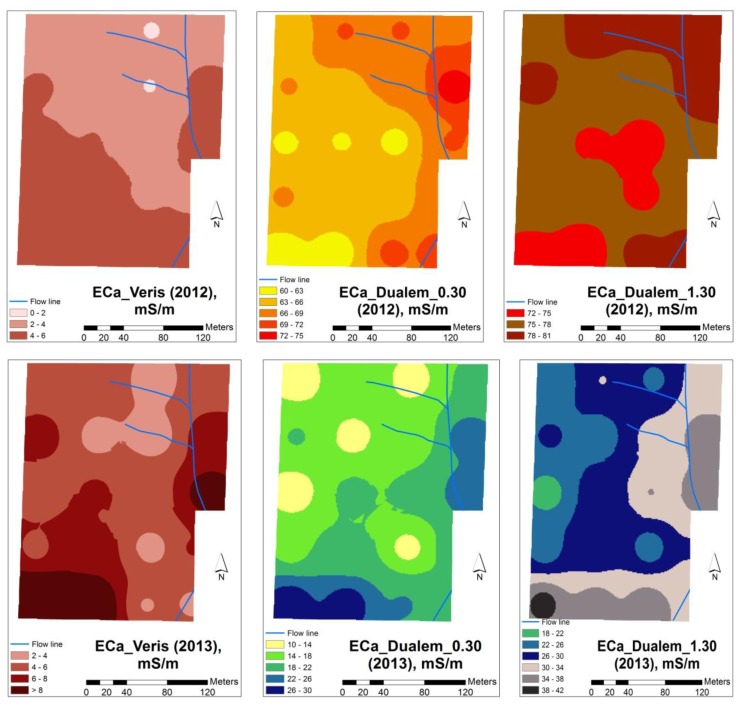
Apparent soil electrical conductivity measured by Veris and DUALEM sensors in the experimental field in February of 2012 (**top**) and February of 2013 (**bottom**).

Figure 7 shows the EC_a_ maps obtained from the measurements with the Veris and DUALEM sensors in February of 2012 and 2013 in the experimental field. In 2012 the EC_a_ spatial patterns obtained by the measurements with the Veris sensor and the measurements with the DUALEM sensor were different, with higher values measured by the Veris sensor observed in the southern slope and around the flow line of the experimental field, while the higher values measured by the DUALEM sensor were observed at the northern slope of the experimental field and in the area around the flow line. In 2013, the EC_a_ spatial patterns obtained by the two sensors had similar tendencies, with higher values measured by both sensors at the southern slope and around the flow line of the experimental field.

### 3.2. Correlation between EC_a_ Measurements

**Figure 8.**
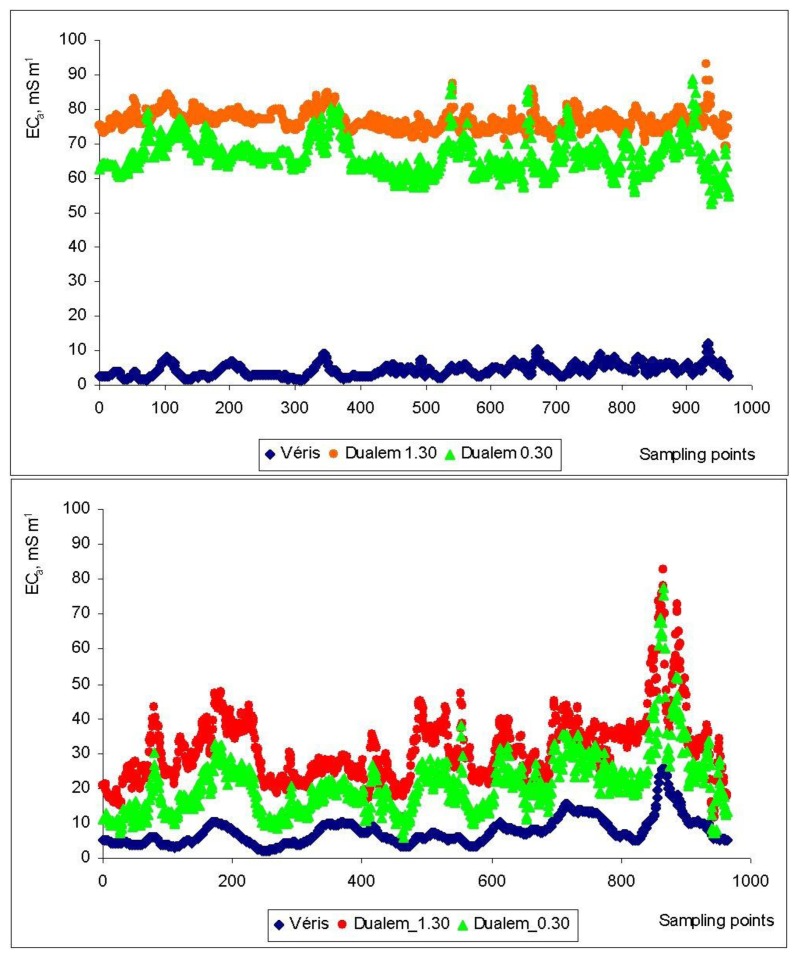
Apparent soil electrical conductivity measured by the Veris and DUALEM sensors in the experimental field in February 2012 (**above**) and February 2013 (**bottom**).

Figure 8 shows the values of EC_a_ measured by the Veris and DUALEM sensors in the 964 points with common geographic co-ordinates in the experimental field, in February 2012 and February 2013. This figure shows similar EC_a_ patterns measured by the two sensors. Sudduth *et al.* [5] and Guo *et al.* [27] mention that although temporal variability exists in EC_a_, the relative pattern of EC_a_ distribution within a field is relatively stable.

Table 2 presents the correlation coefficients between the EC_a_ values measured by Veris and DUALEM sensors, result of the first step of the statistical treatment. Significant correlations between the EC_a_ measurements obtained by these two different sensors can be observed in both measurement dates. The strongest correlations were observed between the measurements of EC_a_ at the two PD (0.30 m and 1.30 m) by the DUALEM sensor (r = 0.919 and r = 0.930, in 2012 and 2013, respectively). Also good correlations were observed between the measurements by the two sensors in 2013 (r = 0.827 and r = 0.675, between Veris and DUALEM 0.30 m and between Veris and DUALEM 1.30 m, respectively). The effect of vegetation on EC_a_ values measured by DUALEM, associated with different wave propagations of the two methods, is evident in 2012, having resulted in a weak correlation between the values measured by Veris and DUALEM sensors.

Figure 8 also shows a different scale of absolute values of EC_a_ measured by the two sensors, which is in contradiction with the observations of Sudduth *et al.* [5] in their comparative study between EMI and ER sensors. Nonetheless, in relative terms, the two sensor measurements clearly show a similar pattern of behavior in both evaluation moments.

The lower values and the smaller variability of the EC_a_ readings by the Veris sensor under conditions of lesser SMC (2012) confirm that reliable operation of the Veris can be more difficult in dry soils due to weak electrical contact between the coulters and the soil [5]. On the other hand, the higher values of EC_a_ measured by the DUALEM sensor under conditions of a more dry soil, but with important and differentiated vegetative development of the pasture (2012) show that this sensor, since it does not contact the soil, is more sensitive to the ground cover than the Veris sensor. In the case where vegetation with varying mass and moisture content is present, the most sensitive area below the DUALEM sensor is filled with vegetation of a potentially large and varying conductivity. According to Sudduth *et al.* [5] instrument response curves for the DUALEM sensors show that the strongest incremental response for the shallow channel is nearest the instrument, and in fact that for the 1 s, approximately 40% of the total response comes from that 0.2 m nearest the instrument. Brevik *et al.* [22] admit that even dry and not very conductive crop residues on the soil surface might alter the soil EC_a_ values obtained with the EMI sensors because these readings are dependent upon the volume of soil included within the sensing depth of the instrument.

### 3.3. Correlation between EC_a_ Measurements and Soil and Pasture Parameters

As a result of the second step of the statistical treatment, Table 3 shows the correlation coefficients between the EC_a_ values measured in the 0–0.30 m PD by the Veris and DUALEM sensors and soil and pasture attributes. Differences were observed between the sensors in the correlations with soil properties in the two experiments. Significant correlation coefficients were found in both surveys (years 2012 and 2013) between the EC_a_ measured by the Veris and SMC, clay, silt, sand (negative), organic matter, pH and phosphorus. The EC_a_ measured by this sensor also shows significant correlations with pasture dry matter yield and pasture moisture content (negative), in 2012 and with potassium and nitrogen, in 2013.

On the other hand, the DUALEM sensor in 2012 only showed significant correlation coefficients with bedrock depth, pasture dry matter yield and pasture moisture content, while in 2013 showed significant correlation coefficients with SMC, silt, pH, phosphorus and potassium. There is a difference in the sign of correlation between the Veris and DUALEM 2012 in the pasture variable: for the Veris, there is a negative correlation and for the DUALEM, there is a positive correlation. This is a key finding regarding the differential effect of the vegetation on the two sensors.

**Figure 9.**
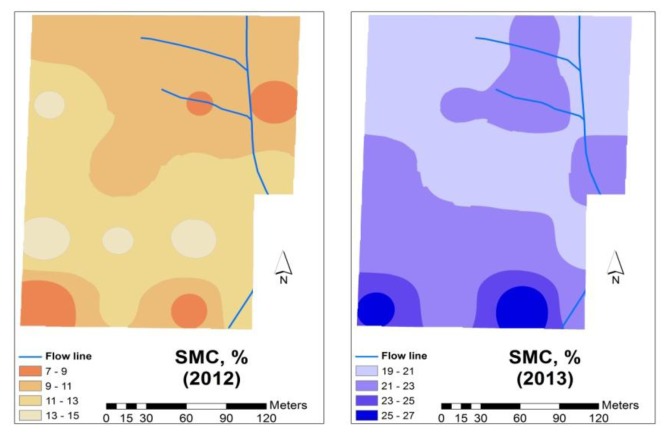
Spatial soil moisture variability in the experimental field.

Figure 9 shows the SMC maps of the experimental field. The low levels of SMC in February 2012, of around 10%, reflect the relatively dry winter of 2011/2012 in the Mediterranean area. On the other hand, in both years (2012 and 2013), the variability of this parameter is relatively small and a tendency for higher SMC values is observed in the southern slope, which corresponds to the higher values of soil clay content (Figure 4) and soil organic matter (Figure 6) but also of smaller bedrock depth (Figure 3, right).

**Figure 10.**
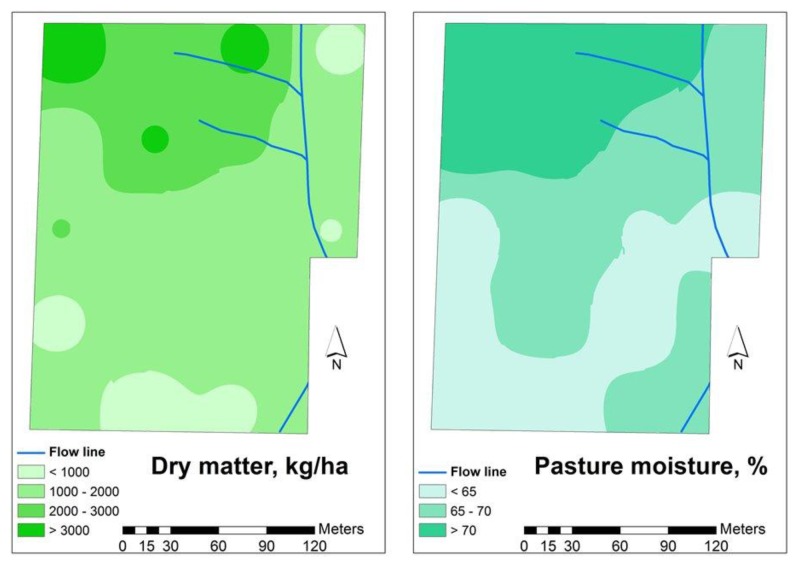
Pasture dry matter yield and pasture moisture content in February of 2012 in the experimental field.

Figure 10 shows pasture dry matter yield and pasture moisture content maps in February 2012, the year in which the pasture was not grazed. Greater development of the pasture and higher moisture content in the pasture can be observed at the northern slope.

### 3.4. Integrated Discussion

The main objective of this study was to compare two apparent soil electrical conductivity (EC_a_) sensors (Veris 2000 XA and DUALEM 1S) for mapping variability of soil properties in a Mediterranean shallow soil. Based on published studies [14], a good correlation was expected between EC_a_ and soil parameters that remain relatively stable throughout the years, such as pH, organic matter content and clay content. Previous works carried out by this team with the DUALEM sensor at the same experimental field [18] showed significant correlations only in the case of soil pH. One objective of this study was to try to clarify if this weak correlation with clay and soil organic matter is specific to the EMI sensor or is similar to ER sensors. The results show that unlike DUALEM, the Veris sensor shows significant correlations with pH, clay and soil organic matter, in both survey dates.

This study also aimed at studying the effect of soil cover vegetation on the EC_a_ measurement by the two types of sensors. It is possible that in the present study and with this type of soil, the pattern of EC_a_ measurements carried out with the DUALEM sensor in February 2012 operating at a height of around 20 cm above the soil surface covered by a dense layer of pasture (with variable moisture content), reflects, in particular, the pasture moisture content. In this case, the relationship between EC_a_ and clay and soil organic matter content may have been confounded by concurrent changes in the pasture moisture content. This is not the case with the measurements carried out with the Veris sensor, since it interacts directly with the soil surface.

Regarding EC_a_ measured by the DUALEM sensor, the results obtained in 2013, with short vegetation and relatively homogeneous development (grazed pasture), show a significant correlation with pH, but not with clay and soil organic matter. One important aspect that might have conditioned the correlation between the relatively stable soil properties and the EC_a_ measured by DUALEM is the presence of shallow soils at the hill tops, where soil depth is quite reduced (very superficial clay horizon) due to erosive processes [18]. At these locations, the sensor was also measuring the EC_a_ of the bedrock, and the results are strongly influenced by the degree of weathering of the bedrock, with an adverse effect on the values of this parameter and probably in a differentiated form by the two measured depths. Similar results were obtained by Bronson *et al.* [6], due to low bulk density of the shallow calcic horizon at their site. Previous studies have shown that soil properties that influence EC_a_ readings are influenced by landscape position [22]. Brevik *et al.* [22] mention that soils located in lower landscape positions tend to have higher clay content and greater water content and, therefore, tend to have higher EC_a_ readings when compared to soils located higher on the landscape. Murphy *et al.* [28] confirm that in dry land farming, there is a tendency for smaller values of SMC in areas located higher on the landscape due to the effect of the greater solar exposure and of more shallow soils at these areas, since water tends to flow and accumulate in the lower areas of the field in response to gradients in gravitational potential energy. Nonetheless, this experimental field showed a tendency for higher SMC values in the southern slope, which correspond to the higher values of soil clay content and soil organic matter but also of smaller bedrock depth. These anomalies of the SMC field distribution might be explained by the large clay content in these areas of the field, since sand drains more rapidly whereas clay retains more moisture [29]. The season of the year in which the soil sampling was done, at mid-winter (February) justify this SMC distribution. For Carroll and Oliver [29] the soil volumetric water content showed smaller spatial variation when the soil was close to saturation (February 2013) than when the soil was drier (February 2012), which influenced the values of EC_a_. This aspect is especially important because, according to Brevik *et al.* [20] EC_a_ techniques have their best mapping potential in fields with soils that exhibit a wide EC_a_ range and will not work well in fields that have soils with fairly uniform electrical properties.

These results confirm that surface topography, due to its effect on the SMC, plays a significant role in influencing spatial EC_a_ variation. Under these conditions, in shallow soils, SMC is the main factor influencing the value of EC_a_. Various authors have observed a linear relationship between SMC and EC_a_ for each level of soil salinity over the range of measured soil moistures [3,30]. Soil moisture estimated from EC_a_ reflects changes in landscape and soil characteristics.

## 4. Conclusions

This study compares two EC_a_ sensors, the Veris 2000 XA and DUALEM 1S, contact and non-contact type, respectively, for mapping soil properties variability in a Mediterranean shallow soil. The work consisted of two surveys carried out under very different situations which are common in pastures: (i) with low SMC and high and differentiated vegetation development (non grazed pasture); and (ii) with high SMC and short and relatively homogeneous vegetation development (grazed pasture).

Despite having observed significant correlations between the EC_a_ measurements obtained by the two sensors at both evaluation moments, these behaved differently in the correlations with the soil properties. The greater temporal stability presented by the Veris sensor in this study, under conditions of wide variation in the SMC and vegetation ground cover, indicates the suitability and the potential of using this sensor for monitoring texture, SMC, organic matter, pH and phosphorus soil content in permanent pastures.

The correlation of EC_a_ measured by the DUALEM sensor with the soil properties was not consistent at the two different situations. These differences between sensors are relevant for the selection of an EC_a_ sensing system for a particular application. For practical purposes, the DUALEM sensor which does not directly touch the soil, can be used in soils covered with vegetation preferably when the crop is not very densely developed.

In order to further evaluate the dynamics of EC_a_ sensors, it is desirable to extend these comparative field trials to other types of soils in the region and different levels of soil vegetation cover and pasture moisture content.

## Figures and Tables

**Table 1.** Range, mean and standard deviation of apparent soil electrical conductivity and others soil parameters of the experimental field.

**Parameter**	**Minimum**	**Maximum**	**Mean**	**SD**
Apparent soil electrical conductivity (EC_a_), mS·m^−1^				
Veris (2012)	1.5	8.3	4.0	1.6
Veris (2013)	2.4	31.1	6.8	4.5
DUALEM 0.30 (2012)	55.8	78.4	66.1	4.5
DUALEM 0.30 (2013)	10.8	65.5	20.0	8.6
DUALEM 1.30 (2012)	70.4	81.8	76.3	2.3
DUALEM 1.30 (2013)	18.3	74.2	30.6	9.4

Others soil parameters				
Bedrock depth, cm	20.0	70.0	44.9	16.2
SMC (2012), %	4.9	14.6	10.7	2.4
SMC (2013), %	18.2	28.1	21.5	2.4
Clay, %	23.4	43.2	34.4	4.9
Silt, %	15.6	27.6	21.7	2.8
Sand, %	31.0	55.4	43.9	5.7
pH	5.6	7.3	6.1	0.3
Organic matter, %	1.3	2.7	1.6	0.2
P_2_O_5_, mg kg^−1^	21.5	261.5	47.2	27.9
K_2_O, mg kg^−1^	58.0	270.5	89.7	24.6
NO_3_, mg kg^−1^	5.0	55.4	16.0	7.0

SMC- Soil moisture content; SD- Standard deviation.

**Table 2.** Correlation coefficients between the EC_a_ data measured by the two sensors.

**EC_a_ Measure**	**Veris (2012)**	**DUALEM 0.30 (2012)**	**DUALEM 1.30 (2012)**	**Veris (2013)**	**DUALEM 0.30 (2013)**	**DUALEM 1.30 (2013)**
Veris (2012)	1	0.211 *	0.259 *	0.265 *	0.261 *	0.195 *
DUALEM 0.30 (2012)		1	0.919 **	0.199 *	0.140 *	0.163 *
DUALEM 1.30 (2012)			1	ns	ns	0.111 *
Veris (2013)				1	0.827 **	0.675 **
DUALEM 0.30 (2013)					1	0.930 **
DUALEM 0.30 (2013)						1

* Correlation is significant with 95% of confidence level;

** Correlation is significant with 99% of confidence level; ns-not significant.

**Table 3.** Correlation coefficients between the EC_a_ data (measured in the 0–0.30 m PD by Veris and DUALEM sensors) and soil and pasture parameters.

**Attributes**	**Veris (2012)**	**Veris (2013)**	**DUALEM 0.30(2012)**	**DUALEM 0.30(2013)**
*Soil*				
Bedrock depth, m	ns	ns	0.643 **	ns
Soil Moisture Content, %	0.365 *	0.379 *	ns	0.338 *
Clay, %	0.516 **	0.262 *	ns	ns
Silt, %	0.303 *	0.315 *	ns	0.323 *
Sand, %	−0.624 **	−0.479 *	ns	ns
Organic matter, %	0.589 **	0.617 **	ns	ns
pH	0.721 **	0.524 **	ns	0.531 **
P_2_O_5_, mg kg^−1^	0.598 **	0.597 **	ns	0.458 *
K_2_O, mg kg^−1^	ns	0.460 *	ns	ns
NO_3_, mg kg^−1^	ns	0.555 **	ns	0.458 *
*Pasture*				
Dry matter yield, kg ha^−1^	−0.682**	-	0.368*	-
Moisture content, %	−0.478*	-	0.420*	-

* Correlation is significant with 95% of confidence level;

** Correlation is significant with 99% of confidence level; ns-not significant.
